# Sportomics suggests that albuminuria is a sensitive biomarker of hydration in cross combat

**DOI:** 10.1038/s41598-022-12079-7

**Published:** 2022-05-17

**Authors:** Luis C. O. Gonçalves, Anibal M. Magalhães-Neto, Adriana Bassini, Eduardo Seixas Prado, Renan Muniz-Santos, Marcio V. A. Verli, Lukas Jurisica, Jaqueline S. S. Lopes, Igor Jurisica, Claudia M. B. Andrade, L. C. Cameron

**Affiliations:** 1 Laboratory of Protein Biochemistry, The Federal University of State of Rio de Janeiro (UNIRIO), Rio de Janeiro, Brazil; 2 Health Sciences Center, University Center of Rio de Janeiro (UNIRJ), Rio de Janeiro, Brazil; 3 Pharmaceutical Assistance, Health Secretary, Angra Dos Reis, Brazil; 4 Health Surveillance, Health Secretary, Itaguaí (PMI), Rio de Janeiro, Brazil; 5 Graduate Program in Health Sciences, Faculty of Medicine, Federal University of Mato Grosso (UFMT), Cuiabá, Brazil; 6 Institute of Biological and Health Sciences, Federal University of Mato Grosso (UFMT), Cuiabá, Brazil; 7 Laboratory for Research in Physical Exercise and Metabolism, Federal University of Alagoas (UFAL), Maceió, Brazil; 8 School of Computer Science, Carleton University, Ottawa, Canada; 9 Health Sciences Center, University Center of Araguaia Valley (UNIVAR), Barra do Garças, Brazil; 10 Osteoarthritis Research Program, Division of Orthopedic Surgery, Schroeder Arthritis Institute-and Data Science Discovery Centre for Chronic Diseases, Krembil Research Institute, University Health Network, Toronto, Canada; 11 Departments of Medical Biophysics and Computer Science, University of Toronto, Toronto, Canada; 12 Institute of Neuroimmunology, Slovak Academy of Sciences, Bratislava, Slovakia; 13 Chemistry Department, Institute of Exact and Earth Sciences, Federal University of Mato Grosso (UFMT), Cuiabá, Brazil

**Keywords:** Biochemistry, Diagnostic markers, Predictive markers, Kidney

## Abstract

We have been using sportomics to understand hypermetabolic stress. Cross Combat (CCombat) has recently been initiated as a high-intensity functional training method inspired by CrossFit. We used a CCombat session to induce metabolic stress and evaluated its effects on hydration and kidney function. Blood samples were collected from 16 elite-level professional male athletes engaged in training sessions over a 96-h protocol. Blood myoglobin increased by ~ 3.5-fold (119 ± 21 to 369 ± 62 nmol/L; *p* = .001) in response to the protocol, returning to the pre-exercise level within 48 h. Furthermore, d-dimer levels increased from 6.5 ± 0.6 to 79.4 ± 21.3 μmol/L (*p* < .001) in response to exercise decreasing during recovery with high variability among the studied athletes. Albuminemia and creatininemia increased ~ 10% and cystatin C increased ~ 240% (1.7 ± 0.1 to 5.7 ± 0.5 mg/L; p < .001; effect size = 2.4) in response to the protocol. We measured albuminuria (HuA) to assess kidney permeability to albumin caused by exercise. HuA increased ~ 16-fold (0.16 ± 0.03 to 2.47 ± 0.41 μmol/L; *p* < .001; effect size = 1.4) in response to exercise, dropping and reaching basal levels during 48 h. Here, we suggest that microalbuminuria can be used as an early, sensitive, easy, and inexpensive biomarker to evaluate hydration status changes during intensive exercise, decreasing chronic impairment in renal function.

## Introduction

Sportomics is a subject-centered holistic method that focuses on sports as the metabolic challenge^1^ (for an editorial and reviews, see Bragazzi 2020; Bassini and Cameron 2014; Sellami et al. 2022)^2–4^. In addition, the use of metabolomics is rapidly gaining space in understanding exercise and sports^5^. We have been utilizing sportomics and other in-field exercise models to understand hypermetabolic stress in health and disease^2,3^. Unlike the studies carried out in the laboratory under controlled conditions, sportomics involves samples collected in the field, enabling us to capture important temporal physiological changes during training and competition. Although in-the-field measurements are inherently less controlled and thus more variable, sportomics leads to reproducible results with high sensitivity^6–8^.

High-intensity functional training (HIFT), like high-intensity interval training (HIIT), is a new training modality that mixes high-intensity exercises with functional movements, and CrossFit (CFit) training has emerged as the most common and growing form of HIFT worldwide^9,10^. It has been proposed that HIIT is the essence of combat sport-specific training^11^. Cross Combat (CCombat), a new method inspired by CFit, has recently been initiated as a training method using specific fighting movements. CCombat is divided into three specific training levels: beginner, intermediate, and advanced. In the beginners' group, the cargo is the body mass itself, whereas at subsequent levels, auxiliary equipment common to traditional CFit is added to fighting movements. Unfortunately, many reports have linked CFit training to severe mechanical injuries or life-threatening conditions, such as rhabdomyolysis^12,13^.

Adequate hydration is a central biological issue, and inadequate fluid intake associated with intensive exercise programs has been associated with harmful conditions, such as rhabdomyolysis and the impairment of renal filtration^14,15^. The first reports of rhabdomyolysis in the modern era occurred during World War II, and some biblical references are related to the disease (for a review, refer to Zager^16^), and hydration can both prevent and treat this condition^17,18^. The evaluation of nitrogenous compounds such as creatinine, urea, and urate is of great value in daily clinical follow-up; however, the elevation of these parameters in the blood is already a consequence of the impairment of renal filtration. To assess kidney function, the experimental gold standard is the measurement of either inulin or iothalamate clearance^19,20^, although it has not been used in clinical practice. In this context, the discovery of physiological early markers of hydration is crucial for hydration assessment during metabolic stress.

The identification of early and reliable biomarkers of acute kidney levels at either the systemic or cellular level is of great interest for both science and clinical applications^21^. In this setting, we hypothesized that a sportomics approach may reveal new markers of hydration and establish a temporal relationship of onset and washout of these substances. For that purpose, in this study, we used a 40 min CCombat protocol to induce metabolic stress studied under a sportomics approach evaluating the effects of CCombat on hydration and kidney function.

## Methods

### Subjects

After an announcement in major fight centers in Rio de Janeiro, we selected 62 elite-level mixed martial arts (MMA) who covered the inclusion criteria. The inclusion criteria were being an adult male (we were trying to avoid hormonal bias due to the nature of the study), having a minimum of 5 years of professional experience in MMA training and competitions with at least 2 years of experience in CCombat training. The exclusion criteria were the use of supplements in the past 2 months that could interfere with the metabolism, and either the use of anabolic aids or having had musculoskeletal system lesions in the last 2 years. We excluded 14 athletes because of the use of different thermogenic agents; 12 for having used steroids hormones or steroid receptors modulators and eight for having different musculoskeletal system lesions. In addition, we excluded ten athletes at the moment of the experiment who were not fasting at the time of the first collection, and two that did not follow the rest described in the protocols. Therefore, after the application of the exclusion criteria, 16 athletes (23–36 years; 30.0 ± 1.3) engaged in this study. The subjects' demographics are described in Table 1 (supplementary information).

### Experimental design

The athletes engaged in high-intensity functional exercise (40 min) in an observational and cross-sectional cohort study. All participants rested for 72 h before the exercise protocol. The CCombat protocol was individually created to achieve an intensity level of 70% repetition maximum, as calculated as previously described^17^. The protocol involved 12 different exercises combined in six modules according to their equivalence.

Anthropometric parameters were measured at various experimental times using a calibrated scale and stadiometer. Additionally, to investigate the consequences of the new patented training method CCombat, we evaluated hemodynamic, metabolic, and hydration parameters in a 40-min section of a designed CCombat protocol.

### Blood sampling

Blood samples were collected at the following times: 2 days before the experiment (− 48 h); 1 day before the experiment (− 24 h); pre-exercise collection (Pre); immediately after exercise (Post); 60 min after the end of exercise (+ 60 min), 120 min after the end of exercise (+ 120 min); 24 h after the end of exercise (+ 24 h); and 48 h after the end of exercise (+ 48 h).

**Figure 1:**
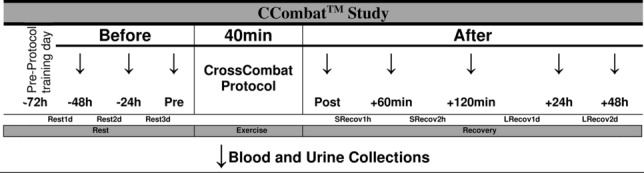
Experimental design. The subjects rested for 3 days before the CrossCombat (CCombat) protocol when samples were collected for 96 h, starting 2 days before (− 48 h; − 24 h) until 2 days after (24 h; 48 h) the exercise protocol (Pre; Post; + 60 min; + 120 min). The CCombat protocol started from 07:00 to 09:00, splitting the athletes to avoid overlapping the venipuncturist and securing immediate sample collections. Advanced-level CCombat training was performed for 40 min. Arrows indicate the eight-blood and urine collections times.

Blood samples were collected from the medial cephalic vein by an experienced venipuncturist. Samples were collected either in the presence or absence of EDTA (Vacutainer^®^, BD, NJ, USA) for hematological or serum analyses, respectively (Fig. 1).

### Blood or serum measurements

Hematocrit was measured using microhematocrit tubes and centrifugation. Serum albumin (HSA), urate, total blood proteins, ALT, AST, GGT, amylase, and alkaline phosphatase were measured using a Piccolo^®^ General Chemistry 13 rotor (Abaxis, CA, USA). Cortisol, myoglobin, d-dimer, CK-MB, and cystatin C were assessed using ichroma alpha^®^ (Boditech Med Inc., Gangwon-do, Korea). Lactate, blood pH (SpH), and bicarbonate (SHCO3^−^) were evaluated using CG4 + cartridges and I-STAT (Abbot-Abbott Point of Care Inc., NJ, USA). CHEM8 + cartridges and I-STAT were used to determine creatinine, glucose, urea, sodium, chloride, and potassium levels.

### Urine measurements

Urine samples were collected at the same time frame as blood samples during the CCombat training protocol. Urine was evaluated using reagent strips for urinalysis (Labor Import, São Paulo, Brazil). Urinary albumin (HuA), urine specific gravity (uSG), pH, ketone, bilirubin, glucose and nitrate were measured with ichroma alpha^®^ (Boditech Med Inc., Gangwon-do, Korea).

### Calculations

Blood osmolality was calculated as described in Worthley et al.^22^. and the estimated glomerular filtration rate was calculated as previously discussed by Inker et al.^23^.

### Statistical analysis

Our sample represents nearly 7% of the elite-level MMA professional athletes' population in Brazil (~ 25% in Rio de Janeiro), making our study relevant for the field.

The Shapiro–Wilk test was applied to verify the distribution of the data. Significance was examined using the Mann–Whitney nonparametric test or *t-*test, as appropriate, based on the data distribution. Additionally, Cohen's dz effect size and statistical power were calculated to analyze the data and identify statistically significant differences^24^.

To produce analyte correlation matrices, raw data were analyzed using a nonparametric Spearman's rank-order correlation from the SciPy Python library (rs, scipy.stats.spearmanr method, ver. 1.4.1). Diagonal entries represent self-correlations, and off-diagonal entries represent direct positive or negative correlations of pairwise analytes (the correlation matrix is symmetric). The discussion focuses only on pairs with rs > 0.5 and a significance of p < 0.05. Data were visualized using the PyPlot Python library (ver. 3.1.2). The color of each cell was then determined by linearly interpolating the correlation (blue for positive; red for negative).

Significance in this study was set at p < 0.05, and data are presented as the average ± standard error (AVG ± SE).

Raw measured data were normalized against Pre measures according to Bassini and Cameron^3^, as necessary for improved interpretation.

### Ethical approval

This study was conducted according to the guidelines laid down in the Declaration of Helsinki, and the experiment met the requirements of research using human subjects (National Health Council, 2012). This study was approved by the Ethics and Research Committee (number 2,230,073) of the Federal University of Mato Grosso (UFMT) and was registered at clinicaltrials.gov (NCT 03522883). The individuals were informed that they could withdraw from the study at any time. Written informed consent was obtained from each subject, who was instructed on the nature of the research and the procedures involved.

## Results

The precise physiological and biochemical effects of the high-impact exercise CCombat remain poorly understood. Therefore, to investigate the consequences of the new patented training method CCombat, we evaluated hemodynamic, metabolic, and hydration parameters in a 40-min section of a designed CCombat protocol (Fig. 1).

### The CCombat protocol induced a transient decrease in volemia

**Figure 2:**
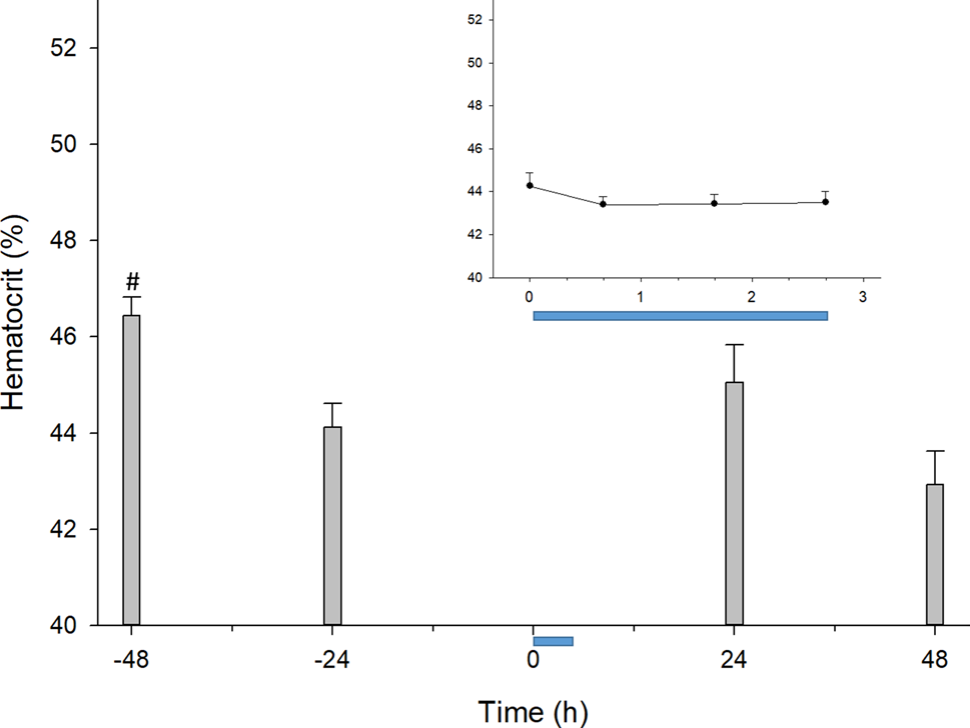
Volemia did not change in response to exercise. #Pre vs*.* − 48 h: *p* = .010; effect size = 1.0; statistical power = 0.94. The blue bar represents the CCombat protocol in scale in both graphs. AVG ± SE.

Blood ranges from 7 to 8% of body mass in mammals, and we assessed changes in hematocrit. To evaluate possible changes in blood volume, we assessed changes in hematocrit. There was a significant decrease in hematocrit at the pre-exercise moment. Hematocrit decreased by approximately 5% during the second rest day (i.e., Rest2d) after the previous training and did not change (ranging from 43 to 45%) in response to exercise (0–40 min, i.e., CCombat Protocol) or short (up to 120 min, i.e., SRecover) or long (up to 48 h, i.e., LRecover) recovery (Fig. 2).

### The CCombat protocol is a high-ATP demand exercise

**Figure 3:**
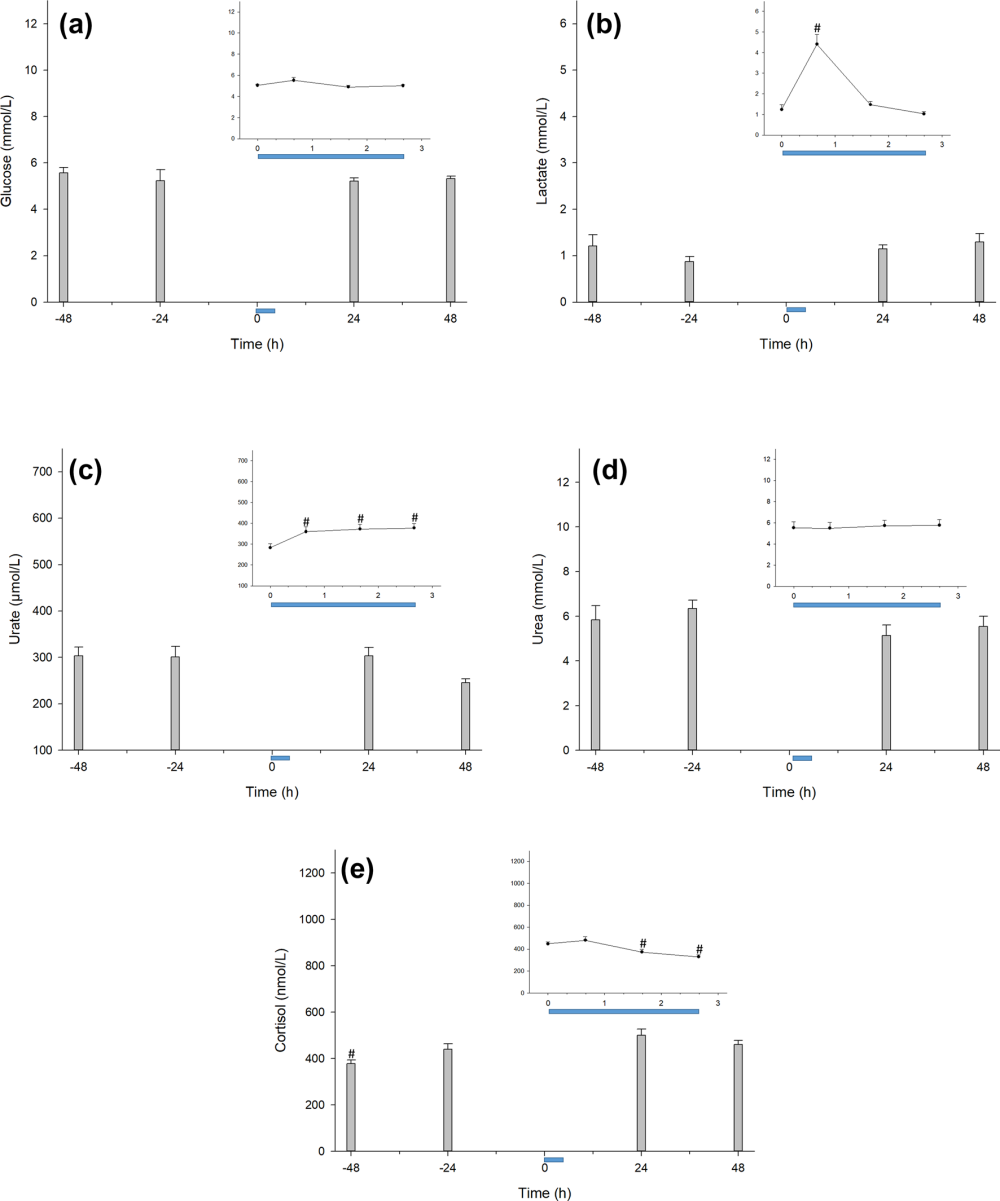
The CCombat protocol promoted an increase in both serum lactate and urate without changing glycemia or uremia. (**a**) Glucose; (**b**) lactate #Pre vs*.* Post: *p* < .001; effect size = 1.9; statistical power = 0.99; (**c**) urate #Pre vs. Post: p = .017; effect size = 0.9; statistical power = 0.92; #Pre vs. + 60 min: *p* = .004; effect size = 1.0; statistical power = 0.97; #Pre vs. + 120 min: *p* = .005; effect size = 1.1; statistical power = 0.98; (**d**) urea and (**e**) cortisol #Pre vs*.* − 48 h: *p* = .010; effect size = 1.0; statistical power = 0.94; #Pre vs*.* + 60 min: *p* = .031; effect size = 0.8; statistical power = 0.83; #Pre vs*.* + 120 min: *p* < .001; effect size = 1.5; statistical power = 0.99. The blue bar represents the CCombat protocol in scale in both graphs. AVG ± SE.

To understand the demands imposed during the CCombat protocol, we investigated some biomarkers of energetic metabolism. Glycemia and uremia did not change before (rest), during, or after (recovery) the exercise protocol (Fig. 3a,d). In contrast, lactate, an indicator of glycolytic pathway demand, increased by approximately 3.7-fold in response to exercise, returning to basal levels during recovery (Fig. 3b). Urate blood concentration can be used as a direct indicator of myokinase activity during exercise, and in this study, urate increased by 27% during CCombat and remained the same during SRecover, reverting to the pre-exercise level during LRecover (Fig. 3c). Conversely, cortisolemia decreased by 27% in response to exercise during SRecover, returning to the basal level during LRecover (Fig. 3e). In addition, we measured a ketonuria tail during rest before the CCombat protocol. Bilirubin was detected in urine on the last day (i.e., 48 h). Nevertheless, there was no measurable presence of glucose or nitrates in the athletes' urine during the whole study (from – 48 to + 48 h, Table 2-supplementary information).

We examined other protein biomarkers to control for other metabolic activities as well as hepatic function. Total blood proteins (TBP), alanine aminotransferase (ALT), aspartate aminotransferase (AST), γ-glutamyl transferase (GGT), amylase, and alkaline phosphatase (AP) did not change during the study (Table 3, supplementary information).

**Figure 4:**
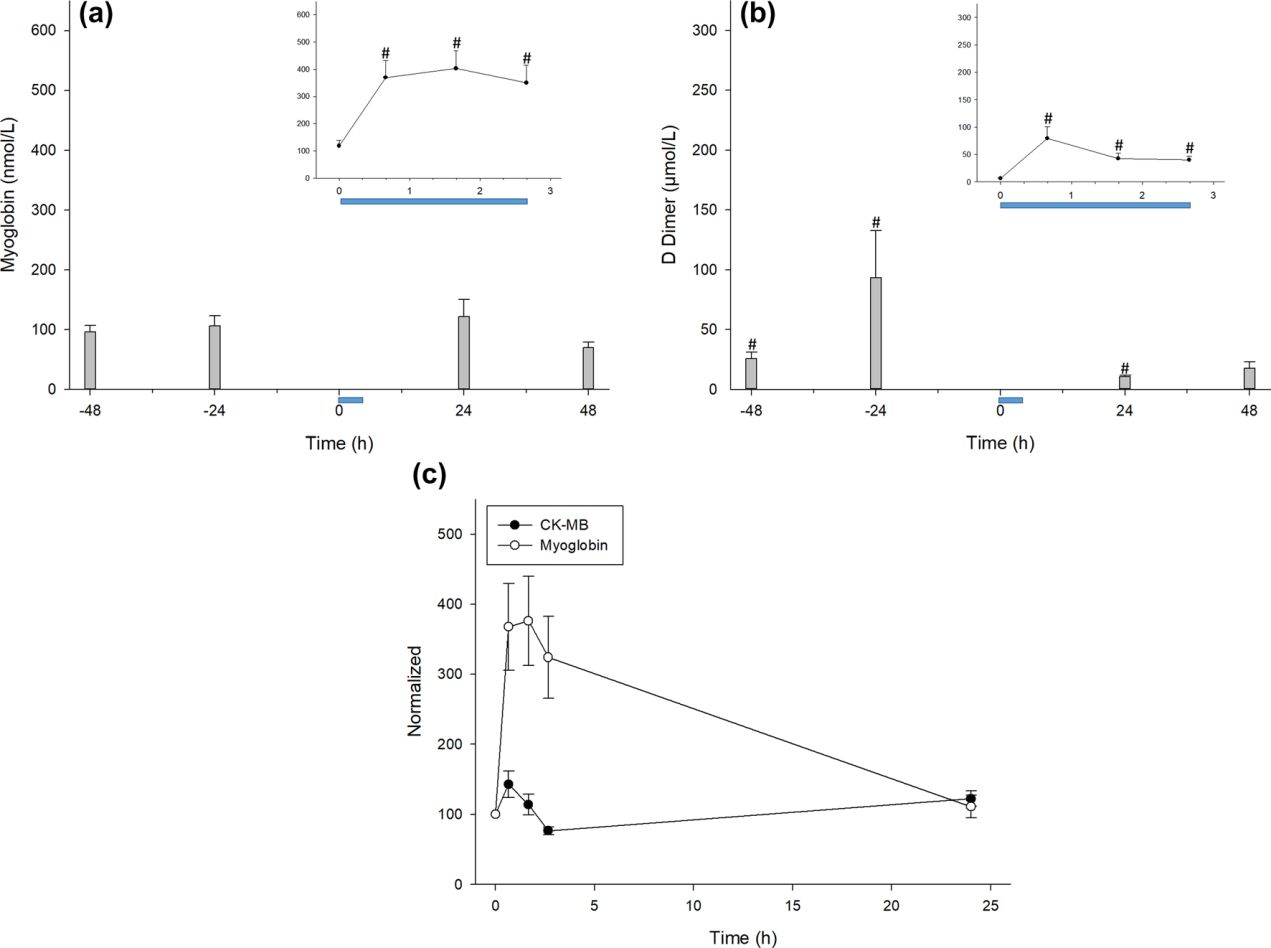
The microinjury muscle biomarkers myoglobin and CK-MB as D-dimer increased in response to the CCombat protocol. (**a**) Myoglobin #Pre vs*.* Post: *p* = .001; effect size = 1.1; statistical power = 0.98; #Pre vs*.* + 60 min: *p* < .001; effect size = 1.2; statistical power = 0.99; #Pre vs*.* + 120 min: *p* < .001; effect size = 1.0; statistical power = 0.96; (**b**) d-dimer #Pre vs*.* − 48 h: *p* < .001; effect size = 0.9; statistical power = 0.94; #Pre vs*.* − 24 h: *p* < .001; effect size = 0.5; statistical power = 0.54; #Pre vs. Post: *p* < .001; effect size = 0.8; statistical power = 0.89; #Pre vs*.* + 60 min: *p* = .002; effect size = 0.9; statistical power = 0.93; #Pre vs*.* + 120 min: *p* < .001; effect size = 1.3; statistical power = 0.99; #Pre vs*.* + 24 h: *p* = .010; effect size = 1.0; statistical power = 0.95 and (**c**) comparison of CK-MB and d-dimer concentrations up to 24 h after the protocol (Pre; Post; + 60 min; 120 min and + 24 h). The blue bar represents the CCombat protocol in scale in both graphs. AVG ± SE.

To understand the effect of CCombat on muscle integrity, we measured myoglobin, d-dimer, and CK-MB levels. Myoglobin in the blood increased by ~ 3.5-fold in response to the CCombat protocol, remaining elevated during SRecover and returning to the pre-exercise level during LRecover (Fig. 4A). Furthermore, d-dimer levels increased in 15 of 16 athletes, ranging from 22 to 5.4-fold in response to exercise and decreasing during recovery (Fig. 4B), and variability in d-dimer behavior was high among the studied athletes. For the results of individual d-dimer changes during the CCombat study, please refer to Table 4 (supplementary information). Due to experimental reasons, we were unable to retrieve CK-MB data for the entire experiment, although CK-MB levels in blood are raised in a very similar manner to myoglobin. In this study, CK-MB increased by up to 20% and decreased to Pre levels during LRecover1d (Fig. 4C).

Glomerular function can be assessed by the kidney's ability to both excrete and reabsorb. In this study, we analyzed electrolyte concentrations in blood to evaluate kidney function and detected no changes in blood concentrations of sodium, chlorine, potassium, glucose, or urea during the entire protocol (Table 5-supplementary information; Fig. 3a,d). In addition, the calculated plasma osmolality did not change during the entire study, a finding confirmed by urine specific gravity measurement (Table 5-supplementary information).

**Figure 5:**
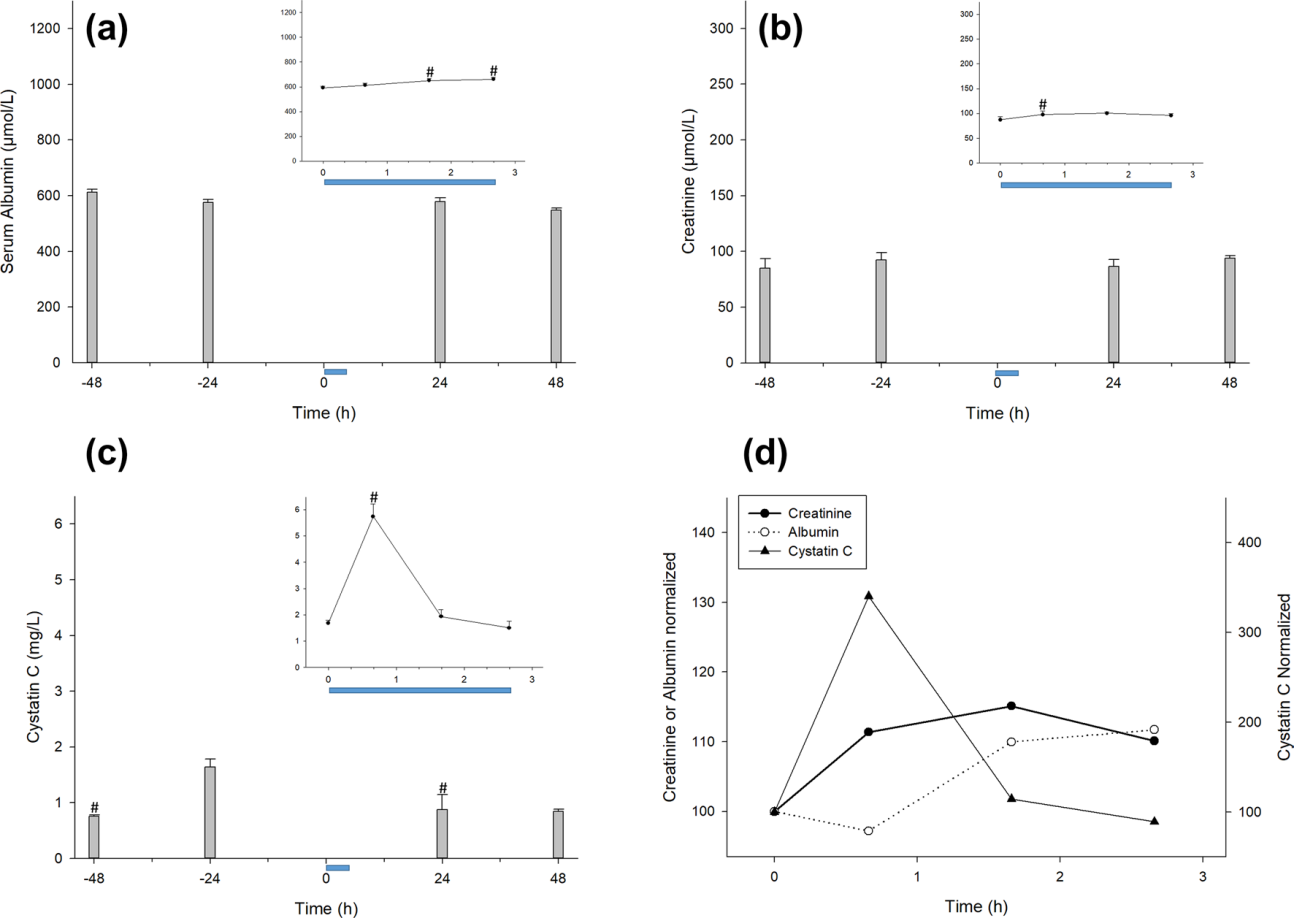
Both serum creatinine and cystatin C increased after the CCombat protocol, followed by serum albumin. (**a**) Serum albumin (HSA) #Pre vs. + 60 min: *p* = .008; effect size = 0.9; statistical power = 0.93; #Pre vs*.* + 120 min: *p* = .001; effect size = 1.2; statistical power = 0.99; (**b**) serum creatinine #Pre vs. Post: *p* = .047; effect size = 0.7; statistical power = 0.80; (**c**) cystatin C #Pre vs*.* − 48 h: *p* < .001; effect size = 2.4; statistical power = 1.00; #Pre vs*.* Post: *p* < .001; effect size = 2.4; statistical power = 1.00; #Pre vs*.* + 24 h: *p* = .007; effect size = 0.8; statistical power = 0.89; #Pre vs*.* + 48 h: *p* < .001; effect size = 2.2; statistical power = 1.00 and (**d**) normalized HSA, creatinine and CysC. The blue bar represents the CCombat protocol in scale in both graphs. AVG ± SE.

As albuminemia (HSA) is another sensitive marker of volemia, we evaluated blood albumin to confirm possible changes in blood volume. HSA increased by 12–14% in SRecover, returning to basal levels during LRecover (Fig. 5a,d). Creatininemia is a well-known indicator of the glomerular filtration rate, and we measured the blood creatinine level to help understand the effects of CCombat on the hemodynamics of athletes. Blood creatinine increased by 11% in response to the CCombat protocol, returning to basal levels within 24 h (Fig. 5b,d). We also followed cystatin C (CysC), a consensus biomarker of glomerular filtration, to assess kidney function during our protocol and found that it increased by 3.4-fold in response to the CCombat protocol, quickly returning to basal levels within the very first hour of recovery (Fig. 5c,d). Additionally, the estimated glomerular filtration rate (eGFR) exhibited a decline in response to exercise, indicating a decrease in filtration (Supplementary Table 5).

**Figure 6:**
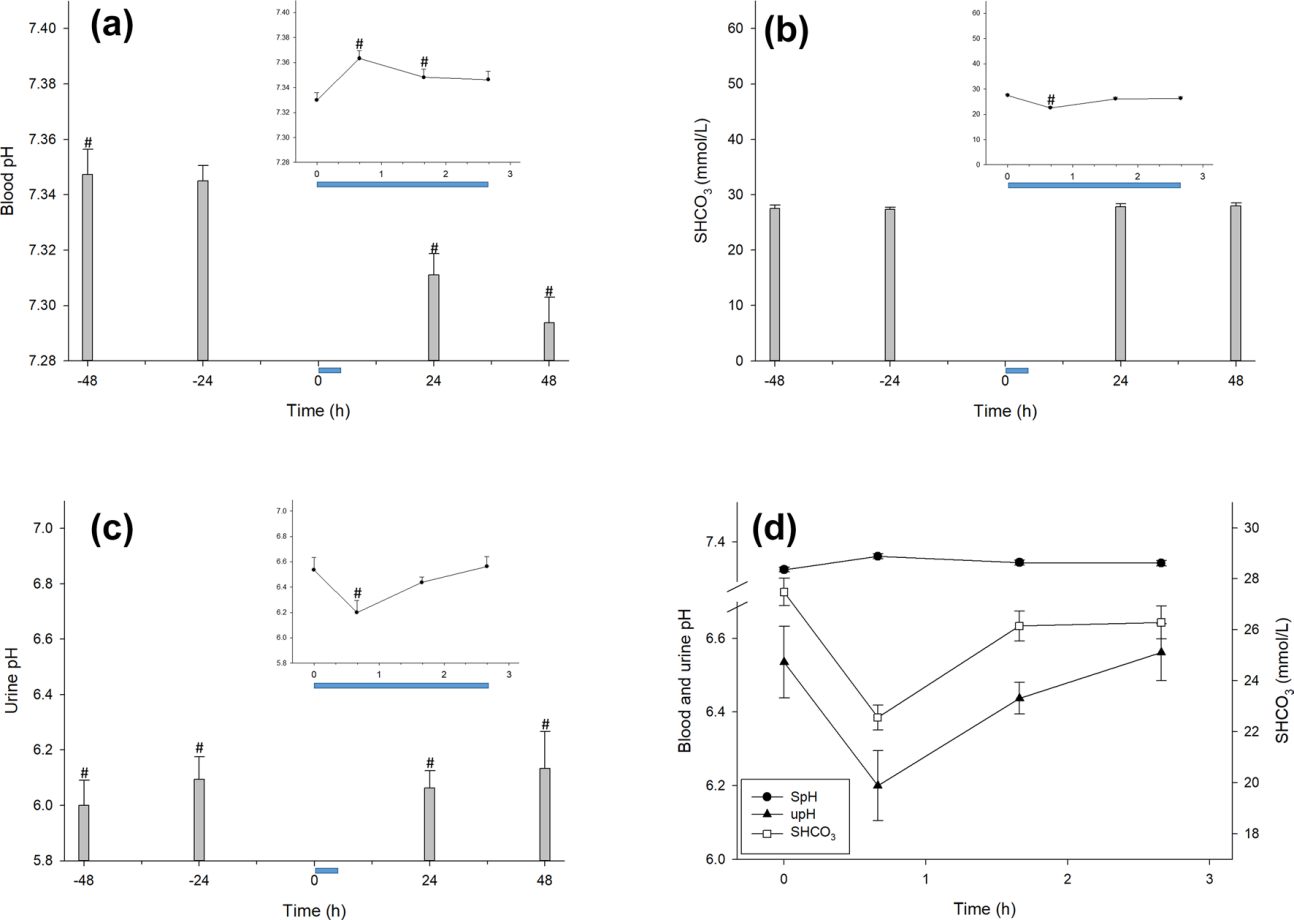
Blood pH did not change physiologically due to the CCombat protocol, although serum bicarbonate and urinary pH decreased. (**a**) Blood pH (SpH) #Pre vs*.* − 48 h: *p* = .046; effect size = 0.5; statistical power = 0.55; #Pre vs*.* Post: *p* < .001; effect size = 1.3; statistical power = 0.99; #Pre vs*.* + 60 min: *p* = .046; effect size = 0.7; statistical power = 0.76; #Pre vs*.* + 24 h: *p* = .038; effect size = 0.6; statistical power = 0.71; #Pre vs*.* + 48 h: p = .002; effect size = 1.1; statistical power = 0.98; (**b**) serum bicarbonate (SHCO3) #Pre vs*.* Post: *p* < .001; effect size = 2.3; statistical power = 1.00; (**c**) urine pH (upH) #Pre vs*.* − 48 h: *p* < .001; effect size = 1.4; statistical power = 0.99; #Pre vs*.* − 24 h: *p* = .002; effect size = 1.2; statistical power = 0.99; #Pre vs*.* Post: *p* = .016; effect size = 0.9; statistical power = 0.92; #Pre vs*.* + 24 h: *p* < .001; effect size = 1.4; statistical power = 0.99; #Pre vs*.* + 48 h: *p* = .014; effect size = 0.8; statistical power = 0.90 and (**d**) comparison of pH values and bicarbonate concentrations during CCombat protocol and short recovery. The blue bar represents the CCombat protocol in scale in both graphs. AVG ± SE.

The lungs and kidneys are the primary organs that regulate blood pH. To understand the effects of the CCombat protocol on blood pH, we measured the pH of both blood (SpH) and urine (upH) based on the concentration of serum bicarbonate (SHCO_3_^−^). Although SpH did not change in response to the CCombat protocol, both SHCO_3_^-^ and upH decreased by 18% and 5% (note that we are referring to pH units, i.e., exponential decay), respectively (Fig. 6a–d).

### The CCombat protocol induces temporary microalbuminuria

**Figure 7:**
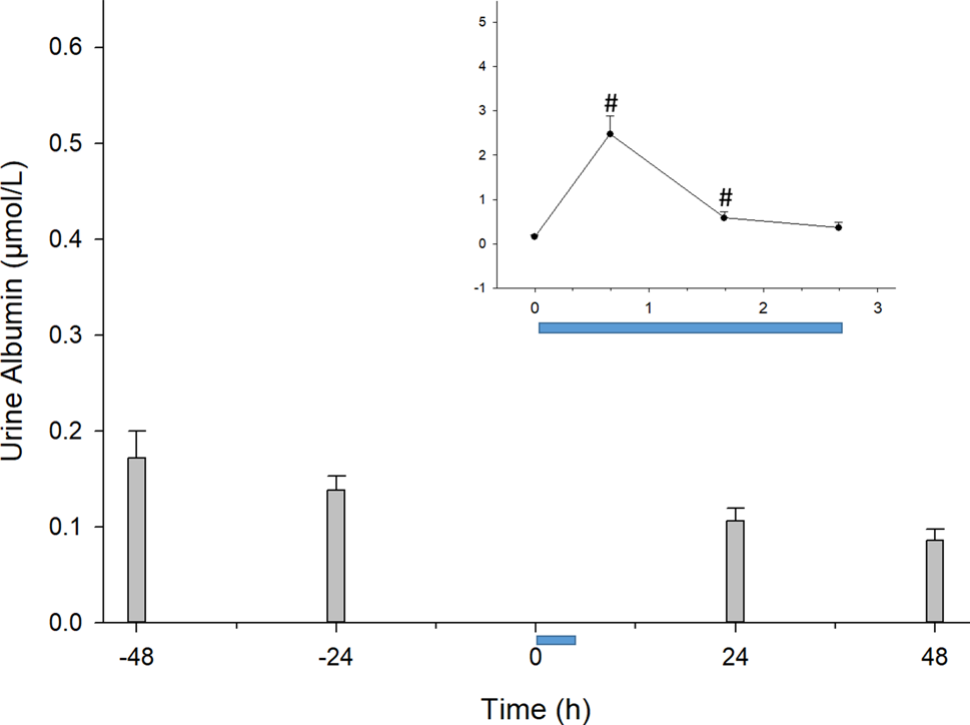
The CCombat protocol led to a 16-fold transient increase in albuminuria. #Pre vs*.* Post: *p* < .001; effect size = 1.4; statistical power = 0.99; #Pre vs*.* + 60 min: *p* < .001; effect size = 0.7; statistical power = 0.81. The blue bar represents the CCombat protocol in scale in both graphs. AVG ± SE.

Blood albumin can change acutely due to exercise primarily because of either water movement from the blood to the interstitium or to a change in glomerular filtration. Accordingly, we measured albuminuria (HuA) as a way of exploring alterations in kidney permeability to albumin caused by exercise. HuA remained constant during rest, with an increase of ~ 16-fold (1600%) in response to exercise, dropping in SRecover and reaching basal levels during LRecover (Fig. 7).

### Integrated analysis

**Figure 8:**
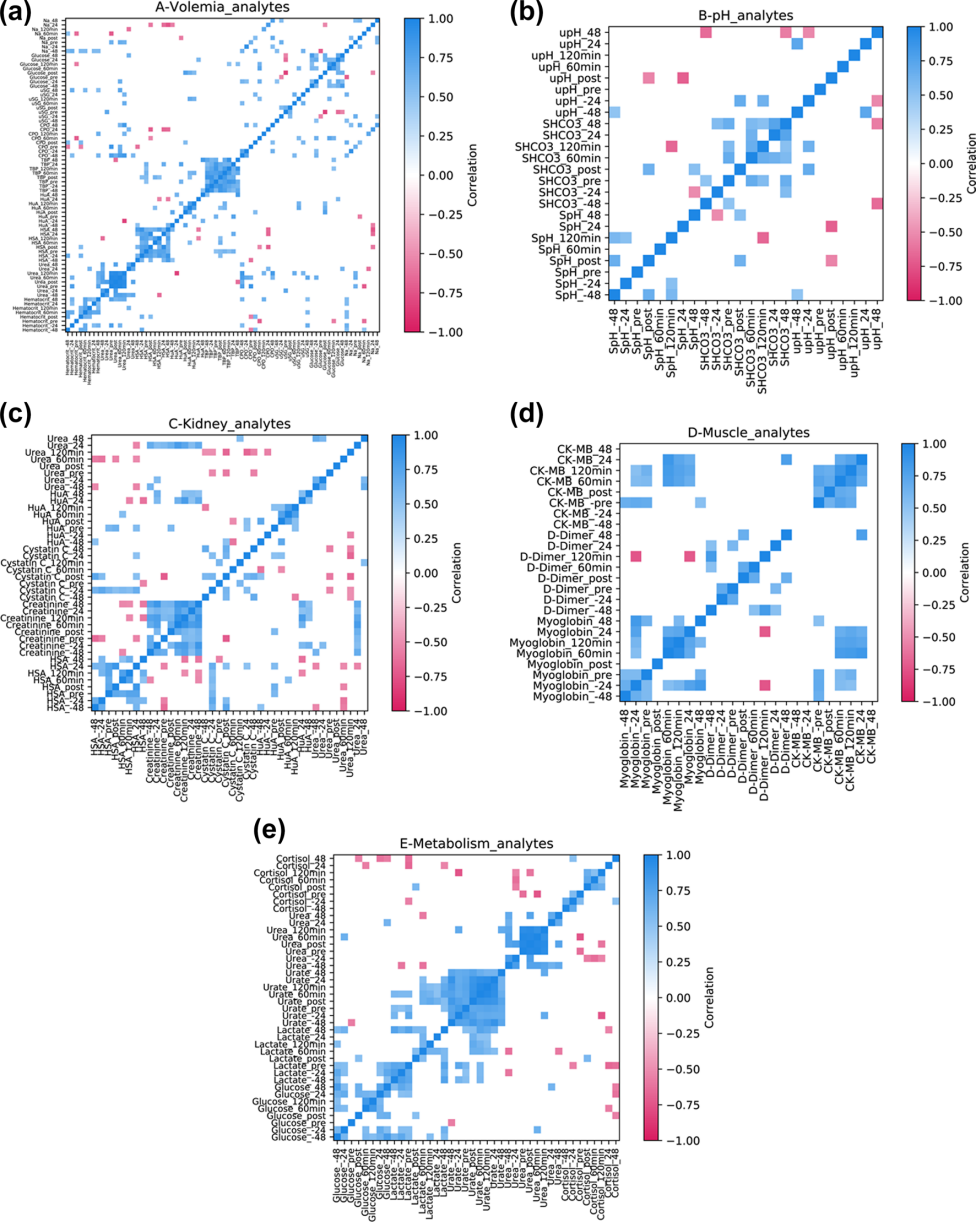
Analyte correlations with the CCombat protocol. Correlation matrices related to (**a**) volemia (hematocrit; urea; serum albumin concentration (HSA); total blood proteins (TBP); sodium; glucose; calculated plasma osmolality (CPO); urine specific gravity (uSG) and urinary albumin (HuA)); (**b**) pH (blood pH (SpH); serum bicarbonate (SHCO3); urine pH (upH)); (**c**) kidney (serum albumin concentration (HSA); serum creatinine; cystatin C; urinary albumin (HuA); urea); (**d**) muscle (myoglobin; d-dimer and CK-MB); (**e**) metabolism (glucose; lactate; urate; urea and cortisol). The discussion focuses only on pairs with rs > 0.5 and a significance of p < 0.05.

For integrated analysis of systemic functions, we built a correlation map of selected analytes. We evaluated analytes correlating with volemia to identify indirect indicators of blood water content. Natremia (− 48 h) and hematocrit (− 48 and − 24 h) changes correlated highly during resting, with r_s_ values of 0.55 and 0.65, respectively (Fig. 8a). In addition, changes in blood pH due to the CCombat protocol (SpH Post) correlated negatively with urinary pH (upH Post, r_s_ − 0.54) and positively with serum bicarbonate (SHCO_3_^−^ Post, r_s_ 0.64). Moreover, SpH and SHCO_3_^-^ displayed a negative correlation (120 min; r_s_ − 0.67) during short recovery (Fig. 8b). The LRecover HuA response correlated highly with creatinine during both SRecover and LRecover; HuA at 48 h and creatinine at 60 min showed a similar trend, but the correlation was not significant (r_s_ 0.48, p = 0.06). CysC Post and creatinine Pre correlated negatively (r_s_ − 0.71); HuA Post correlated negatively with CysC at 60 min (r_s_ − 0.50) and positively with CysC at 24 h (r_s_ 0.53) (Fig. 8c). The parallel response of CK-MB and myoglobin to the CCombat protocol can be seen in the first 24 h of recovery, and these proteins showed a correlation during SRecover until LRecover at 24 h (r_s_ 0.66–0.86) (Fig. 8d). To understand the metabolic response to the CCombat protocol, we performed correlation analysis of metabolism markers and cortisol, in which glucose Post and lactate Post showed a positive correlation (r_s_ 0.52); lactate SRecover and LRecover correlated positively with urate Post and SRecover (r_s_ 0.50–0.74), and urea Post correlated negatively with glucose Post (r_s_ − 0.50) (Fig. 8e).

## Discussion

In this study, we used a CCombat protocol to induce metabolic stress, and we evaluated the effects on hydration and kidney function under a sportomics approach. Our study is observational, and we present results from in-field measurements across various metabolic events. In discussing the main results, we focus herein on correlations at r_s_ > 0.5 (Spearman's rank-order correlation) with a significance of p < 0.05. We emphasize that even small changes in protein expression or metabolite concentration and their correlation with a marker can represent a biologically meaningful signal, thus recognizing the importance of some results that show only low correlations. Additionally, it should be stressed that the correlation of parallel events does not imply causation.

Hematocrit is a well-known indicator of hemoconcentration due to many erythrocytes (RBCs) in the blood, and it has been used as a tool for hydration follow-up in exercise^25,26^. The athletes in our study rested for 72 h before the CCombat protocol; this rest period was preceded by a day of noncontrolled intense training. As a result, we detected a decrease in hematocrit during the beginning of rest (Rest 2d). As natremia and hematocrit changes correlated highly during rest, we attribute the decline in hematocrit in Rest2d to the restoration of hydration after the previous intense training, as highlighted in the correlation map and as previously described^27^.

We did not observe changes in hematocrit during the CCombat protocol or recovery, suggesting no major changes in volemia in these athletes. As the athletes had free access to hydration after the CCombat protocol, the outflow of water measured by way of an increase in albumin was quickly compensated for by postexercise hydration.

During our CCombat protocol, there was no change in either glycemia or uremia. However, lactate and urate increased in response to exercise. Such an increase in urate may be used as an indirect indicator of myokinase activity increase in response to exercise^3,28^. In our study, the increase in both urate and lactate concentrations was associated with uremia maintenance. Analyses of changes in metabolite concentrations and correlations with glucose, lactate, urate, and urea suggest that the high energetic demand of this cyclic exercise has a significant contribution to myokinase activity. In addition, the decrease in ketonuria during the rest indicates that the previous training section had an important metabolic contribution to fatty acid oxidation.

Our CCombat protocol induced a highly correlated increase in myoglobin and CK-MB during the first 24 h of recovery. CK-MB and myoglobin are consensus biomarkers of muscle microinjuries^29,30^. Variance in D-dimer concentrations among the studied athletes was high, presenting different kinetics and increases. d-Dimer is a well-known biomarker of coagulation^31,32^. Because all of our athletes were professionals and actively competing, the mechanical and traumatic muscle stress caused by training and competition was expected to be highly variable. Additionally, one athlete presented detectable bilirubinuria (at + 48 h) and high d-dimer levels on the previous day (at + 24 h). Hence, we chose to show individualized d-dimer behavior due to the different mechanical or traumatic triggers causing variations in blood d-dimer levels. We believe that further individualized analyses of the d-dimer response are imperative for a correct understanding of the impact of the CCombat protocol on this biomarker.

In this study, an increase in creatinine and CysC (~ 10% and 250%) was measurable in blood at the end of the exercise program. Creatinine is a metabolite of creatine, and creatininemia is a well-accepted indicator of glomerular filtration rate (GFR). Nevertheless, due to the systemic effect of intensive exercise on muscle damage, an increase in creatininemia can be challenging to interpret^33^. In addition to creatinine, CysC is a protein that reflects GFR and has the advantage of not being affected by diet, muscle loss, or muscle disease^19^. Overall, levels of creatinine and CysC in blood do not change linearly, and relatively small increases in their concentrations represent significant decreases in GFR^33^. Therefore, it is reasonable to postulate that during our CCombat protocol, the athletes experienced a reduction in GFR due to either lack of hydration or increased sudoresis (or their combination), causing an elevation in blood concentrations of both creatinine and CysC.

Furthermore, albuminemia increased (~ 10%) during the short recovery period and returned to basal levels during the long recovery period. The major blood protein albumin is also used as a volemia biomarker in health, disease, and exercise assessments^34,35^. In this study, creatinine and albumin increased at the same ratio in response to exercise, with a slight delay for the latter. The increase in albuminemia just after the rise in both creatinine and CysC in blood appears to be related to a last escape of water from the blood in response to exercise^35^. Nonetheless, our results did not show changes in the major electrolytes in the blood. Therefore, it is possible to infer that the sodium concentration was maintained due to the shortness of the CCombat protocol (40 min), precluding measurable changes in blood osmolality. Additionally, the increase in albuminemia was not linked to an increase in blood osmolality due to the high molecular weight of the protein.

We also measured a slight increase in blood pH in response to exercise. In parallel, we observed a decrease in both serum bicarbonate and urinary pH. Even without measuring CO_2_ exchange, these data suggest that athletes maintain blood pH with minimal variation during heavy CCombat training. As CCombat is a cyclic exercise, the data suggest that H^+^ production during CCombat training can be buffered adequately.

Microalbuminuria (urinary albumin ranging from 0.45 to 4.5 μmol during a 24-h period; MA) occurs because of changes in glomerular filtration due to either changes in intraglomerular pressure or structural damage to glomeruli^36^. MA is also an early sign of kidney impairment^37,38^. Since the work of Leube in 1878, intense exercise has been known to cause albuminuria^39–41^. Microalbuminuria in exercise appears to be primarily linked to the renin–angiotensin–aldosterone system and can be prevented by captopril, depending on training and exercise volume and intensity. Similarly, isradipine can influence the presence of albumin in urine^42–44^. In a previous study, the B-blocker bisoprolol reduced resting albuminuria without affecting exercise-induced permeability of the protein^45^. In our study, we excluded subjects with a history of diabetes, hypertension, or the use of drugs known to impair or protect against albumin leakage.

Urine provides an alternative noninvasive matrix for the discovery of biomarkers^5,46^. "Albumin in urine is a critical marker in the assessment of kidney damage, but the diagnosis can be even more precise if the values of CysC and serum creatinine level are also associated with it"^47^. In general, calculation of the eGFR using serum creatine and CysC in addition to age provides an accurate prediction of GFR^23,48^. Our results revealed a transient decrease in eGFR, which can be explained by an acute loss of water inside the vascular compartment. Acute hypohydration during exercise may lead to either transient kidney hypoperfusion or microglomerular injury, followed by glomerular filtration impairment, as previously described^49^. Our results showed a significant transient increase in microalbuminuria as great as 16-fold combined with GFR impairment. Additionally, our data indicated that the CCombat protocol responses of albuminuria and CysC were similar in shape and highly correlated. Furthermore, increases in albuminuria and CysC were followed by a transient rise in albuminemia. Thus, it is possible that impaired GFR demonstrated by the biomarkers creatinine and CysC leads to cellular level damage causing transient albumin permeability and albuminuria.

Here, we suggest that microalbuminuria can be used as an early, sensitive, easy, and inexpensive biomarker to evaluate hydration status changes during intensive exercise, decreasing chronic impairment in renal function. Due to the similarity between CCombat training and CFit, microalbuminuria can be considered an essential marker for subsequent activity in both training platforms. Since either inulin or iothalamate clearance is regarded as the gold standard for measuring GFR^20^, further confirmation of our findings may be performed using one of these molecules to further understand the phenomena described herein.

### Limitations

This study did not measure ambient temperature, relative humidity or fluid replacement during the protocol. These parameters may be evaluated in future studies. Due to the infield nature of our protocol, extrapolation of our results should be prevented.

### Practical applications

Considering these findings, we recommend that urinary evaluation by albuminuria can be both an easy and vital marker for measuring hydration changes and could be used in either sports or exercise science to aid human performance and to study renal function.

## Conclusions

To the best of our knowledge, this study is the first scientific investigation of CCombat. Taking the results together, it is possible to explore how we might indirectly measure lack of hydration under a sportomics approach as a model. Additionally, the results suggest that under these conditions, albuminuria can be used as an easy and vital marker for evaluating hydration status changes during intensive exercise, such as CCombat. As a practical application we suggest that albuminuria can be a cheap an easy biomarker to follow up athletes’ hydration status during training and competition, and more that can be useful in occupational risk professional assessment as an example in firefighters.

## Supplementary Information

Supplementary Information.

## Data Availability

All data generated or analyzed during this study are included in this published article (and its supplementary information files).

## References

[1] Bongiovanni T (2019). Sportomics: Metabolomics applied to sports. The new revolution?. Eur. Rev. Med. Pharmacol. Sci..

[2] Bragazzi NL, Khoramipour K, Chaouachi A, Chamari K (2020). Toward sportomics: Shifting from sport genomics to sport postgenomics and metabolomics specialties promises, challenges, and future perspectives. Int. J. Sports Physiol. Perform..

[3] Bassini A, Cameron LC (2014). Sportomics: Building a new concept in metabolic studies and exercise science. Biochem. Biophys. Res. Commun..

[4] Sellami M, Elrayess MA, Puce L, Bragazzi NL (2021). Molecular big data in sports sciences: State-of-art and future prospects of OMICS-based sports Sciences. Front. Mol. Biosci..

[5] Khoramipour K (2021). Metabolomics in exercise and sports: A systematic review. Sports Med..

[6] Bessa A (2008). High-intensity ultraendurance promotes early release of muscle injury markers. Br. J. Sports Med..

[7] Gonçalves LC (2012). A sportomics strategy to analyze the ability of arginine to modulate both ammonia and lymphocyte levels in blood after high-intensity exercise. J. Int. Soc. Sports Nutr..

[8] Resende NM (2011). Metabolic changes during a field experiment in a world-class windsurfing athlete: A trial with multivariate analyses. OMICS.

[9] Claudino JG (2018). CrossFit overview: Systematic review and meta-analysis. Sports Med. Open.

[10] Ben-Zeev T, Okun E (2021). High-intensity functional training: Molecular mechanisms and benefits. Neuromol. Med..

[11] Franchini E (2020). High-intensity interval training prescription for combat-sport athletes. Int. J. Sports Physiol. Perform..

[12] Feito Y, Burrows EK, Tabb LP (2018). A 4-year analysis of the incidence of injuries among CrossFit-trained participants. Orthop. J. Sports Med..

[13] Adhikari P, Hari A, Morel L, Bueno Y (2021). Exertional rhabdomyolysis after CrossFit exercise. Cureus.

[14] Rollo I (2021). Fluid balance, sweat Na^+^ losses, and carbohydrate intake of elite male soccer players in response to low and high training intensities in cool and hot environments. Nutrients.

[15] Gomes JH (2020). Acute leucocyte, muscle damage, and stress marker responses to high-intensity functional training. PLoS One.

[16] Zager RA (1996). Rhabdomyolysis and myohemoglobinuric acute renal failure. Kidney Int..

[17] Cabral BMI, Edding SN, Portocarrero JP, Lerma EV (2020). Rhabdomyolysis. Dis. Mon..

[18] Mao HD (2021). Exertional rhabdomyolysis in newly enrolled cadets of a military academy. Muscle Nerve.

[19] Ferguson TW, Komenda P, Tangri N (2015). Cystatin C as a biomarker for estimating glomerular filtration rate. Curr. Opin. Nephrol. Hypertens..

[20] White CA (2021). Simultaneous glomerular filtration rate determination using inulin, iohexol, and. Kidney Int..

[21] Ai JY (2021). The effect of acute high-intensity interval training on executive function: A systematic review. Int. J. Environ. Res. Public Health.

[22] Worthley LI, Guerin M, Pain RW (1987). For calculating osmolality, the simplest formula is the best. Anaesth. Intensive Care.

[23] Inker LA (2012). Estimating glomerular filtration rate from serum creatinine and cystatin C. N. Engl. J. Med..

[24] Cohen J (2013). Statistical Power Analysis for the Behavioral Sciences.

[25] Stewart IB, McKenzie DC (2002). The human spleen during physiological stress. Sports Med..

[26] Nolte HW, Nolte K, Hew-Butler T (2019). Ad libitum water consumption prevents exercise-associated hyponatremia and protects against dehydration in soldiers performing a 40-km route-march. Mil. Med. Res..

[27] Barley OR, Chapman DW, Abbiss CR (2020). Reviewing the current methods of assessing hydration in athletes. J. Int. Soc. Sports Nutr..

[28] Bassini A (2013). Caffeine decreases systemic urea in elite soccer players during intermittent exercise. Med. Sci. Sports Exerc..

[29] Reichel T (2020). Reliability and suitability of physiological exercise response and recovery markers. Sci. Rep..

[30] Nieman DC (2020). Effects of whey and pea protein supplementation on post-eccentric exercise muscle damage: A randomized trial. Nutrients.

[31] Johnson ED, Schell JC, Rodgers GM (2019). The D-dimer assay. Am. J. Hematol..

[32] Zadow EK, Kitic CM, Wu SSX, Fell JW, Adams MJ (2018). Time of day and short-duration high-intensity exercise influences on coagulation and fibrinolysis. Eur. J. Sport Sci..

[33] Pasala S, Carmody JB (2017). How to use… serum creatinine, cystatin C and GFR. Arch. Dis. Child Educ. Pract. Ed..

[34] Anderson L (2020). Precision multiparameter tracking of inflammation on timescales of hours to years using serial dried blood spots. Bioanalysis.

[35] Miller GD, Teramoto M, Smeal SJ, Cushman D, Eichner D (2019). Assessing serum albumin concentration following exercise-induced fluid shifts in the context of the athlete biological passport. Drug Test Anal..

[36] Futrakul N, Sridama V, Futrakul P (2009). Microalbuminuria—a biomarker of renal microvascular disease. Ren Fail..

[37] Doi K (2012). Mild elevation of urinary biomarkers in prerenal acute kidney injury. Kidney Int..

[38] Markus MRP (2018). Prediabetes is associated with microalbuminuria, reduced kidney function and chronic kidney disease in the general population: The KORA (Cooperative Health Research in the Augsburg Region) F4-Study. Nutr. Metab. Cardiovasc. Dis..

[39] Lillehoj EP, Poulik MD (1986). Normal and abnormal aspects of proteinuria. Part I: Mechanisms, characteristics and analyses of urinary protein. Part II: Clinical considerations. Exp. Pathol..

[40] Castenfors J (1977). Renal function during prolonged exercise. Ann. N Y Acad. Sci..

[41] Leube WV (1878). Ueber die ausscheidung von eiweiss im harn des gesunden menschen. Arch. Pathol. Anat. Physiol. Klin. Med..

[42] Esnault VL (1991). Captopril but not acebutolol, prazosin or indomethacin decreases postexercise proteinuria. Nephron.

[43] Aldigier JC (1993). Angiotensin-converting enzyme inhibition does not suppress plasma angiotensin II increase during exercise in humans. J. Cardiovasc. Pharmacol..

[44] Giustina A, Bossoni S, Macca C, Romanelli G (1993). Isradipine decreases exercise-induced albuminuria in patients with essential hypertension. Ren. Fail.

[45] Fauvel JP (1990). Cardiovascular reactivity to and renal impact of stress and exercise: Effects of bisoprolol. J. Cardiovasc. Pharmacol..

[46] Pintus R (2021). Sportomics in professional soccer players: Metabolomics results during preseason. J. Sports Med. Phys. Fitness.

[47] Vittori LN, Romasco J, Tarozzi A, Latessa PM (2021). Urinary markers and chronic effect of physical exercise. Methods Mol. Biol..

[48] Inker LA (2021). New creatinine- and cystatin C-based equations to estimate GFR without race. N. Engl. J. Med..

[49] Wołyniec W, Ratkowski W, Renke J, Renke M (2020). Changes in novel AKI biomarkers after exercise. A systematic review. Int. J. Mol. Sci..

