# Supplementary material for: Sportomics suggests that albuminuria is a sensitive biomarker of hydration in cross combat
Source: Sci Rep. 2022 May 17;12:8150. doi: 10.1038/s41598-022-12079-7 (PMC9114005; doi:10.1038/s41598-022-12079-7)
Supplement: Supplementary file 1 — Supplementary Information. [file 41598_2022_12079_MOESM1_ESM.docx]

**Table 1. Subjects’ demographics.**

|  | Median | Min | Max |
| --- | --- | --- | --- |
| Age (years) | 29 | 23 | 41 |
| Mass (Kg) | 78.5 | 59.0 | 108.0 |
| Height (m) | 1.78 | 1.68 | 1.84 |
| BMI (Kg/m^2^) | 25.5 | 20.2 | 33.0 |

**Table 2. Ketonuria decreased during the rest and did not change in response to the CCombat Protocol.** Average ± SEM. Non-detectable (ND).

|  | -48h | -24h | Pre | Post | +60min | +120min | +24h | +48h |
| --- | --- | --- | --- | --- | --- | --- | --- | --- |
| Ketones (mmol/L) | 1.56±0.75 | 0.63±0.43 | ND | ND | ND | ND | ND | ND |
| Bilirubin (mmol/L) | ND | 0.06±0.06 | ND | ND | ND | ND | ND | 0.03±0.03 |
| Glucose (mmol/L) | ND | ND | ND | ND | ND | ND | ND | ND |
| Nitrite (mmol/L) | ND | ND | ND | ND | ND | ND | ND | ND |

**Table 3.** **Selected protein biomarkers of hepatic function and others metabolic activities did not change during the protocol**. Average ± SEM. Total Blood Protein (TBP); alanine aminotransferase (ALT); aspartate aminotransferase (AST); gamma-glutamyltransferase (GGT); Amylase (AMS) and Alkaline phosphatase (ALP).

|  | -48h | -24h | Pre | Post | +60min | +120min | +24h | +48h |
| --- | --- | --- | --- | --- | --- | --- | --- | --- |
| TBP (g/L) | 82.4±1.3 | 78.1±1.4 | 79.1±1.6 | 81.1±1.3 | 82.6±1.7 | 81.8±1.5 | 79.7±1.7 | 77.8±1.2 |
| ALT (U/L) | 30.6±2.7 | 30.1±2.6 | 28.5±2.6 | 31.0±2.6 | 30.9±2.5 | 30.3±2.4 | 30.2±2.7 | 25.5±1.6 |
| AST (U/L) | 42.5±3.8 | 41.0±3.3 | 41.2±3.5 | 44.3±3.7 | 44.7±3.5 | 45.1±3.7 | 44.1±5.0 | 32.9±2.0 |
| GGT (U/L) | 20.7±2.4 | 19.9±2.6 | 19.7±2.4 | 19.3±2.6 | 20.9±2.4 | 21.7±2.5 | 19.4±2.5 | 19.6±2.6 |
| AMS (U/L) | 54.2±4.6 | 54.1±5.3 | 59.6±8.6 | 57.6±8.1 | 55.1±6.8 | 54.1±6.2 | 59.8±6.2 | 66.5±8.9 |
| ALP (U/L) | 68.7±4.6 | 67.1±5.6 | 68.2±5.6 | 67.7±4.3 | 68.7±4.8 | 66.3±4.6 | 71.1±5.1 | 72.8±5.9 |

**Table 4.** **The CCombat Protocol induced a decrease in the estimated glomerular filtration rate without changes in electrolytes concentrations.** Average ± SEM. Blood concentration of sodium (Na); chloride (Cl); potassium (K); calculated plasma osmolality (PO) and urine specific gravity (SG); estimated glomerular filtration rate (eGFR).

|  | -48h | -24h | Pre | Post | +60min | +120min | +24h | +48h |
| --- | --- | --- | --- | --- | --- | --- | --- | --- |
| Na (mmol/L) | 140.8±0.2 | 140.2±0.3 | 138.4±0.6 | 139.4±0.4 | 139.1±0.4 | 138.5±0.5 | 139.2±0.3 | 140.2±0.3 |
| Cl (mmol/L) | 101.4±0.4 | 102.2±0.6 | 102.8±0.6 | 103.5±0.5 | 101.9±0.6 | 102.9±0.5 | 102.4±0.7 | 101.9±0.6 |
| K (mmol/L) | 3.9±0.1 | 3.9±0.1 | 3.9±0.1 | 3.7±0.1 | 3.9±0.1 | 3.9±0.1 | 3.9±0.1 | 4.0±0.1 |
| PO (mosmol/Kg) | 296.8±0.9 | 296.0±0.9 | 289.7±1.3 | 293.0±0.8 | 291.1±0.8 | 289.8±0.9 | 291.4±1.2 | 295.4±0.8 |
| SG (Kg/m^3^) | 1.026±1x10^-3^ | 1.025±1x10^-3^ | 1.026±1x10^-3^ | 1.027±1x10^-3^ | 1.023±1x10^-3^ | 1.024 ± 2x10^-3^ | 1.024±1x10^-3^ | 1.025±1x10^-3^ |
| eGFR (ml/min/1.73m^2^) | 112.4 ± 2.8 | 71.6 ± 4.9 | 68.8 ± 4.4 | 29.1 ± 2.5 | 67.3 ± 7.6 | 87.4 ± 12.8 | 154.6 ± 22.7 | 108.8 ± 4.1 |

**Table 5.** **There was a wide variation in the temporal patterns of D-Dimer concentrations among the athletes.** The individualized concentrations of D-Dimer are shown during the CCombat™ Study. Due to experimental reasons, we were unable to retrieve the Athlete’s 2 D-Dimer data. Not Available (NA).

|  | -48h | -24h | Pre | Post | +60min | +120min | +24h | +48h |
| --- | --- | --- | --- | --- | --- | --- | --- | --- |
|  | **D-Dimer (μg/L)** | | | | | | | |
| Athlete 1 | 378.6 | 10000 | 162.2 | 606.6 | 1416.6 | NA | 187.6 | 122.8 |
| Athlete 2 | NA | NA | NA | NA | NA | NA | NA | NA |
| Athlete 3 | 212.4 | 2558.1 | 153.8 | 135.2 | 178.9 | 270.9 | 161.6 | 204.9 |
| Athlete 4 | 450.5 | 1254.2 | 168.7 | 4814.2 | 733.5 | 776.7 | 269.5 | 877.2 |
| Athlete 5 | 1377.8 | 2058.0 | 142.4 | 173.9 | 621.8 | 920.5 | 274.4 | 84.0 |
| Athlete 6 | 503.9 | 4194.6 | 118.6 | 1808.2 | 660.3 | 801.5 | 239.3 | 133.2 |
| Athlete 7 | 969.8 | 1145.4 | 143.3 | 3266.1 | 2183.3 | 1022.2 | 234.0 | 669.8 |
| Athlete 8 | 67.6 | 121.9 | 104.4 | 834.0 | 113.2 | 272.8 | 58.8 | 50 |
| Athlete 9 | 736.7 | 569.7 | 72.6 | 1130.1 | 835.7 | 1304.3 | 199.5 | 355.7 |
| Athlete 10 | 386.1 | 644.3 | 77.5 | 751.1 | 616.7 | 1184.2 | 121.4 | 99.7 |
| Athlete 11 | 217.9 | 273.5 | 50.0 | 1036.9 | 119.5 | 232.3 | 61 | 135.8 |
| Athlete 12 | 167.0 | 347.6 | 131.1 | 224.3 | 394.2 | 319.1 | 293.4 | NA |
| Athlete 13 | 319.6 | 89.0 | 50.0 | 145.5 | 109.8 | 410.9 | 50.0 | NA |
| Athlete 14 | 268.4 | 216.5 | 125.8 | 150.8 | 50.0 | 428.5 | 179.91 | NA |
| Athlete 15 | 460.9 | 267.3 | 97.2 | 2318.4 | 1831.6 | 1417.7 | 259.4 | NA |
| Athlete 16 | 68.8 | 132.0 | 54.0 | 2890.0 | 1024.8 | 220.6 | 143.9 | NA |
